# Gradient Evolution of Body Colouration in Surface- and Cave-Dwelling *Poecilia mexicana* and the Role of Phenotype-Assortative Female Mate Choice

**DOI:** 10.1155/2013/148348

**Published:** 2013-09-24

**Authors:** David Bierbach, Marina Penshorn, Sybille Hamfler, Denise B. Herbert, Jessica Appel, Philipp Meyer, Patrick Slattery, Sarah Charaf, Raoul Wolf, Johannes Völker, Elisabeth A. M. Berger, Janis Dröge, Konstantin Wolf, Rüdiger Riesch, Lenin Arias-Rodriguez, Jeanne R. Indy, Martin Plath

**Affiliations:** ^1^Evolutionary Ecology Group, Johann-Wolfgang-Goethe University of Frankfurt, Max-von-Laue-Street 13, 60438 Frankfurt am Main, Germany; ^2^Department of Biology and Ecology of Fishes, Leibniz-Institute of Freshwater Ecology and Inland Fisheries, Müggelseedamm 310, 12587 Berlin, Germany; ^3^Department of Animal and Plant Sciences, University of Sheffield, Western Bank, Sheffield S10 2TN, UK; ^4^División Académica de Ciencias Biológicas, Universidad Juárez Autónoma de Tabasco (UJAT), 86150 Villahermosa, TAB, Mexico

## Abstract

Ecological speciation assumes reproductive isolation to be the product of ecologically based divergent selection. Beside natural selection, sexual selection via phenotype-assortative mating is thought to promote reproductive isolation. Using the neotropical fish *Poecilia mexicana* from a system that has been described to undergo incipient ecological speciation in adjacent, but ecologically divergent habitats characterized by the presence or absence of toxic H_2_S and darkness in cave habitats, we demonstrate a gradual change in male body colouration along the gradient of light/darkness, including a reduction of ornaments that are under both inter- and intrasexual selection in surface populations. In dichotomous choice tests using video-animated stimuli, we found surface females to prefer males from their own population over the cave phenotype. However, female cave fish, observed on site via infrared techniques, preferred to associate with surface males rather than size-matched cave males, likely reflecting the female preference for better-nourished (in this case: surface) males. Hence, divergent selection on body colouration indeed translates into phenotype-assortative mating in the surface ecotype, by selecting against potential migrant males. Female cave fish, by contrast, do not have a preference for the resident male phenotype, identifying natural selection against migrants imposed by the cave environment as the major driver of the observed reproductive isolation.

## 1. Introduction

Environmental gradients can impose divergent selection on populations living along them [1]. Not only can this drive adaptive trait divergence among populations [2, 3], but it can also foster the evolution of reproductive isolation barriers [4–6]. Natural selection can prevent interbreeding of locally adapted populations if (a) immigrants exhibit reduced viability in the habitat type they are not adapted to [7–9], (b) immigrants are unable to complete their reproductive life cycle under the changed environmental conditions [10], or (c) natural selection acts against hybrids with intermediate phenotypes [11]. Moreover, sexual isolation may occur if poorly adapted individuals have a disadvantage in intra- and intersexual competition [12–14], for example, if migrants are at a disadvantage during mate choice [8, 15, 16].

Populations living in extreme environments are of particular interest to evolutionary ecologists. Generally, habitats are considered extreme if certain physical or chemical features of the environment are outside of the range usually experienced by a species and if organisms colonizing this particular habitat type experience an initial reduction in fitness [18, 19]. For example, environments can be extreme due to the presence of toxins and toxicants, like hydrogen sulphide (H_2_S) [20, 21]. H_2_S is acutely toxic to most metazoans because it inhibits aerobic respiration due to its interference with mitochondrial respiration and blood oxygen transport while simultaneously leading to extreme hypoxia in the water [20, 21].

Even perpetual darkness prevalent in cave ecosystems can represent an extreme condition for otherwise surface-dwelling organisms [10, 17]. Darkness interferes with visually-mediated communication and navigation, and so alternative modes of communication and orientation are under strong selection in caves [22–24]. Cave animals are widely used model organisms to study the evolutionary effects of permanent darkness on adaptive trait divergence like improvement of nonvisual senses (e.g., [22, 23, 25, 26]). Traits that become dispensable under lightless conditions (like body pigmentation and the visual system), on the other hand, are reduced convergently in a variety of cave-living taxa like crustaceans [27] and teleost fishes [28, 29], resulting in what is often referred to as the “troglomorphic phenotype” [30]. 

One system characterized by the simultaneous action of two extreme selective forces is located near the southern Mexican village of Tapijulapa [17, 31, 32]. Here, populations of the live-bearing fish *Poecilia mexicana* inhabit environments characterized by all possible combinations of two extreme environmental factors: a sulphidic cave (Cueva del Azufre), a nonsulphidic cave (Cueva Luna Azufre), a sulphidic surface river (El Azufre), and several nonsulphidic, normoxic rivers and creeks [17]. This has led to pronounced phenotypic divergence in several behavioural (e.g., [13, 33–37]), dietary [38, 39], female and male life-history [40–42], morphological [2, 43], and physiological traits [44, 45]. Nevertheless, photoreceptors in cave and surface-dwelling fish appear to be functionally unchanged because spectral sensitivities are virtually identical [46]. Thus, cave populations still have functional, albeit smaller, eyes [46–48] 

**Figure 1:**
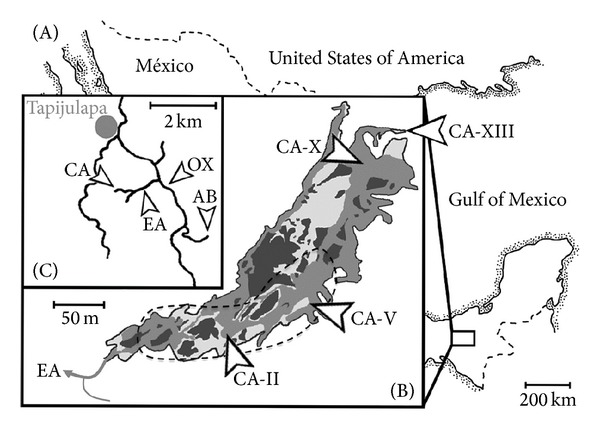
*Map of our study area.* (A) Location of our study area near Tapijulapa, in Tabasco, Mexico. (B) Detail map of the Cueva del Azufre showing the outflow into the El Azufre (EA) and the three different cave chambers investigated (CA-II; CA-V; CA-X). (C) The sites Arroyo Bonita (AB), El Azufre (EA), Rio Oxolotan (RO), and the Cueva del Azufre (CA) were included in the present study.

The Cueva del Azufre provides a small-scale environmental mosaic (Figure 1). While the front-most chambers (like chambers II and V [31]) exhibit moderate H_2_S concentrations (ca. 32 *μ*M [17]) and receive dim light through skylights, chamber X harbours some of the largest sulphide springs within the cave and, accordingly, has high H_2_S concentrations (up to 320 *μ*M [17]), when skylights are absent. Finally, the permanently dark rearmost cave chamber XIII is located upstream of the uppermost sulphide springs and thus representing the only aquatic microhabitat within the cave that does not contain H_2_S. It is separated from all other cave chambers by a small waterfall (~1.6 m height). Thus, up to chamber X, H_2_S-concentrations and light intensity are characterized by a more or less gradual increase or decrease, respectively. 

Surface habitats also differ in their light regimes. The sulphidic (23–41 *μ*M H_2_S [17]) El Azufre creek is turbid due to the presence of sulphates (like gypsum, CaSO_4_) and colloidal sulphur that are generated during oxidation of dissolved H_2_S [17]. Increased turbidity leads to a decrease in available ambient light and a shift in spectral composition as compared to normoxic clear-water streams [49] which could have strong effects on visual perception and thus also mating behaviour [50–52].

 Already in the first description of the cave and its inhabitants by Gordon and Rosen [31] a morphological cline within cave *P. mexicana* was reported, with a gradual decrease in eye size, caudal peduncle depth, and numbers of scales from the front of the cave to the rearmost cave chamber. The gradual change in eye size was subject to numerous investigations [43, 47, 48]. Another study found *P. mexicana* from chamber V, which receives dim light through skylights, to exhibit opsin (*sws*, *rh2*, and *lws*) gene expression similar to conspecifics from surface populations, whereas expression of the same genes was down-regulated in fish from the completely dark chamber X. These differences persist over multiple generations if fish are bred in the laboratory even when kept under daylight conditions, thus pointing to a strong heritable component [53]. On top of that, a gradual change in neuroanatomical structures can be seen. The dimension of the optic tectum gradually decreases from the front chambers to chamber X [54]. 

These clines in morphometric and anatomical characters were attributed to either the presence or absence of light (e.g., assumed for eye size, opsin genes, and optic tectum dimensions) or the presence or absence of H_2_S (e.g., assumed for head size). However, no study to date has provided empirical data on the evolution of body colouration in this system and asked whether a comparable cline in body colouration exists, even though cave-dwelling *P. mexicana* have repeatedly been described as being pale [10, 31, 55–58]. Parzefall [55] provided a qualitative description of surface-dwelling *P. mexicana*. Body colouration in the female sex is reported as a cryptic beige while colouration in the male sex is highly variable, with dominant *P. mexicana *males being more conspicuous in body colouration, showing black vertical bars on the body along with yellowish to orange colour patterns on the margins of the dorsal and anal fins. Subordinate (mostly smaller-bodied) males, however, are more cryptically coloured, with only faint or no vertical bars and little to no orange fin margins.

The lack of a quantitative evaluation of colour differences between *P. mexicana* ecotypes is even more surprising because colour ornaments are known to play a central role in poeciliid communication and mate choice [59–64]. Females of the Trinidadian guppy (*Poecilia reticulata*) are well known to prefer males with high concentrations of carotenoid pigments in the orange spots on their flanks [59]. Carotenoid content in the yolk of cave *P. mexicana* ova, as approximated by egg yellowness, is much lower than in the ova of surface fish [58], so adult cave fish are predicted to also have reduced carotenoid-based colour patterns. In the current study, we therefore investigated male body colouration along the light/dark gradient on a quantitative basis, while including populations from the clear water surface stream Arroyo Bonita and the sulphidic surface stream El Azufre as well as three cave chambers of the Cueva del Azufre (chambers II, V, and X; Figure 1).

In the Cueva del Azufre system, gene flow is strongly reduced between populations with different ecological backgrounds despite the absence of physical migration barriers, and even fish living in different cave chambers are to some extent genetically differentiated [2, 48]. Some degree of unidirectional gene flow from the sulphidic Cueva del Azufre towards the sulphidic El Azufre is detectible, however [48, 65]. Reciprocal translocation experiments confirmed the predicted low viability in fish translocated from non-sulphidic to sulphidic habitats but also from sulphidic into non-sulphidic water [8, 16]. The latter can be explained by exposure to constant hypoxia resulting in the downregulation of cellular oxidative stress protection mechanisms [66]. However, reciprocal translocation experiments between the surface (El Azufre) and the Cueva del Azufre did not find increased mortality rates [8, 67], and other reproductive isolation barriers must account for the observed reduction of gene flow between El Azufre and the Cueva del Azufre. Aquatic heteropterans (*Belostoma *sp.), for example, prey more on immigrant than resident fish (both inside the cave and in the nearby El Azufre), probably because maladapted sensory systems hamper the escape responses of migrant fish [68], and differences in rates of bird predation may play an even more important role in surface waters [69]. 

But why is there weak—albeit traceable—unidirectional gene flow from the cave populations to the El Azufre population? For centuries the Zoque indigenous people have conducted a fertility ceremony (“La Pesca”) during the holy week before Easter, in which they use barbasco plant roots (*Lonchocarpus *sp., Fabaceae) that contain the fish-toxin rotenone to poison cave fish. The anaesthetized fish are drifted outside the cave where they are then collected and eaten [44]; not all sedated fish are captured though, and some recovering individuals might still be able to reproduce. However, this raises the question of why gene flow is surprisingly low despite this massive perturbation every year through the flush of fish out of the cave? We propose that the divergent evolution of colour patterns in cave fish presents a candidate trait that provides cave fish in the surface habitats with a disadvantage, for example, in intersexual selection. 

In our current study we asked whether surface females from the clear water stream Arroyo Bonita and from the sulphidic El Azufre show a preference for resident males over the cave phenotype. We used video animated males to minimize an effect of behavioural differences between ecotypes. Sulphide-adapted cave fish first have to migrate (either actively or passively) through the sulphidic El Azufre to reach non-sulphidic waters such as the Arroyo Bonita. El Azufre females are more likely to face cave-adapted males, and theory predicts stronger conspecific mate preference in populations that inhabit the same—or geographically adjacent—habitats ([70], but see [71] for seasonal variation in conspecific preferences). Hence, we predicted El Azufre females to have stronger preferences for resident male phenotypes over males showing the cave phenotype than females from Arroyo Bonita.

To investigate the second possible migration route, that is, from the surface into the Cueva del Azufre, we conducted another experiment and asked whether cave females discriminate against immigrating El Azufre males in favour of their own males in the absence of visual communication. We evaluated female preferences in the cave using infrared light observation and experimentally allowed females to perceive nonvisual stimuli from the males [25, 35, 72]. Previous laboratory experiments found Cueva del Azufre females to prefer large-bodied over smaller-sized males [25, 35] and well-nourished over malnourished males under dark conditions [73]. The ability to exercise mate choice in darkness has been partly attributed to an enhanced mechanosensory lateral line system with widened pores of the head canal system [74], but also chemoperception plays a role [72]. Based on the virtual absence of gene flow from the El Azufre into the Cueva del Azufre [48, 65] we hypothesized that female cave mollies prefer resident over migrant males.

## 2. Material and Methods

### 2.1. Origin of Test Fish and Preparation of Colour Photos

Male *P. mexicana* for the analysis of body colouration were collected using seines or dip nets in August/September 2011. We sampled surface males in the Arroyo Bonita (AB), a small, sulphide-free tributary to the Río Oxolotán (RO), and El Azufre (EA), a sulphidic creek similar in size and structure to AB (Figure 1). The sample point of the EA population was located directly at the outflow of the Cueva del Azufre (CA), in which we collected fish from chambers II, V, and X.

**Figure 2:**
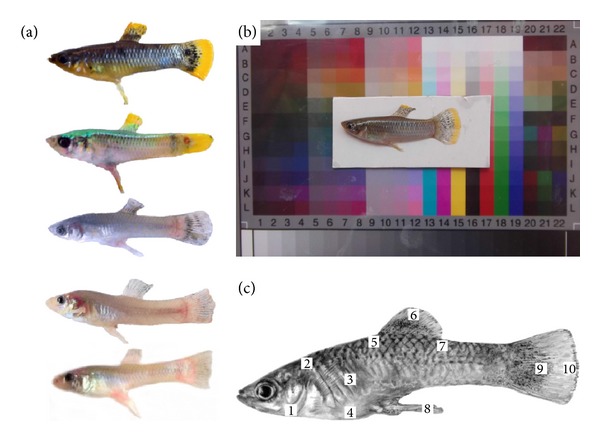
*Male colour phenotypes, calibration plate, and regions on the body and on fins examined in this study.* (a) Examples of *P. mexicana* male colour phenotypes found in the Arroyo Bonita (AB), the El Azufre (EA), and Cueva del Azufre (CA) chambers II, V, and X (from top to bottom). (b) Example of an AB male photographed on the colour calibration plate. (c) Schematic view of the 10 regions for which L*a*b* colour values were determined. The size of the squares represents 1/35 of the individual male's standard length.

To quantify body and fin colouration, wild-caught males were anesthetized directly upon collection using MS222 to ensure that melanophores would be relaxed; that is, maximally opened and full colouration was measured [61]. Anesthetized individuals were laid on a laminated white piece of paper, which in turn was placed on a laminated colour calibration plate (IT8.7/2 LaserSoft Imaging, ID no. R051025; Figure 2(b)) and photographed from centrally above (at approximately 30 cm distance) using a Fujifilm “Finepix AX250 14MP” digital camera; photographs were initially stored as jpeg files (with RGB colour space) but converted to L*a*b* (CIELAB) colour space and stored as.psd files. All photographs were taken while avoiding direct sunlight to minimize reflections.

Surface females used in the mate choice experiments were lab-reared descendants of wild-caught fish captured during various field trips to the study area. We used RO fish, which in population genetic analyses were indistinguishable from the AB population [65]. Test fish came from large, randomly out-bred stocks that were maintained in aerated and filtered 150–200-l aquaria at 27–29°C. Test fish were naïve with respect to the cave phenotype. Artificial light was provided during a 12** **:** **12 hrs light** **:** **dark cycle in addition to natural daylight entering the room through several windows. Fish were fed twice daily *ad libitum* with TetraMin flake food and frozen chironomid larvae. 

#### 2.1.1. Measurement of Male Body Colouration

Images of males were analysed in random order (across and within sites). Prior to all colour measurements, each image was standardized to the colours of the calibration plate according to the manufacturer's instructions. The three coordinates of the L*a*b* (CIELAB) colour space represent the lightness of a colour (where L* = 0 represents black and L* = 100 represents white), its position between green and red/magenta (a* = −150 represents green while a* = +100 represents magenta) and its position between blue and yellow (b* = −100 represents blue and b* = +150 represents yellow). By using the gradation curve modulation in Adobe Photoshop CS5, we calibrated the squares L13 to L19 as well as the white and black squares of the calibration plate (Figure 2(b)) in each photograph to the provided L*a*b* values. This procedure ensured each photograph to have the same standard colouration and thus allowed for a quantitative comparison of individual colour differences. As *P. mexicana* do not have distinct colour spots but a more or less fluent colouration, we decided to measure the colouration at 10 different body regions (Figure 2(c)). We determined the mean colouration of a square parcel (1/35 of the fish's SL; Figure 2(c)) using the Blur/Average option of Adobe Photoshop CS5. For each photographed fish, standard length and body height (max. distance from dorsal to ventral) was also taken. In total, 91 pictures of males from all sites were analysed (Table 1).

#### 2.1.2. Statistical Analysis

**Figure 3:**
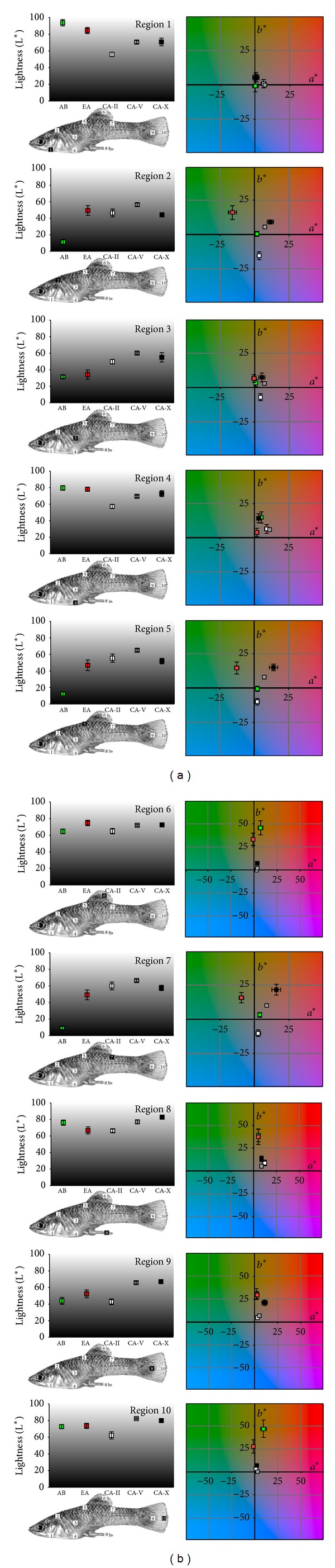
*Colouration of P. mexicana males from 5 different populations.* Shown are means (±SE) for L*-values (left side) as well as a* and b*-values (right side) of the 10 different body regions examined (respective region is indicated at left side as black square with white number). For the background of the a*b*-graph, L*-values were set at 50 throughout. Arroyo Bonita (AB, green square), El Azufre (EA, red square), Cueva del Azufre chamber II (CA-II, white square), chamber V (CA-V, grey square), and chamber X (CA-X, black square).

**Figure 4:**
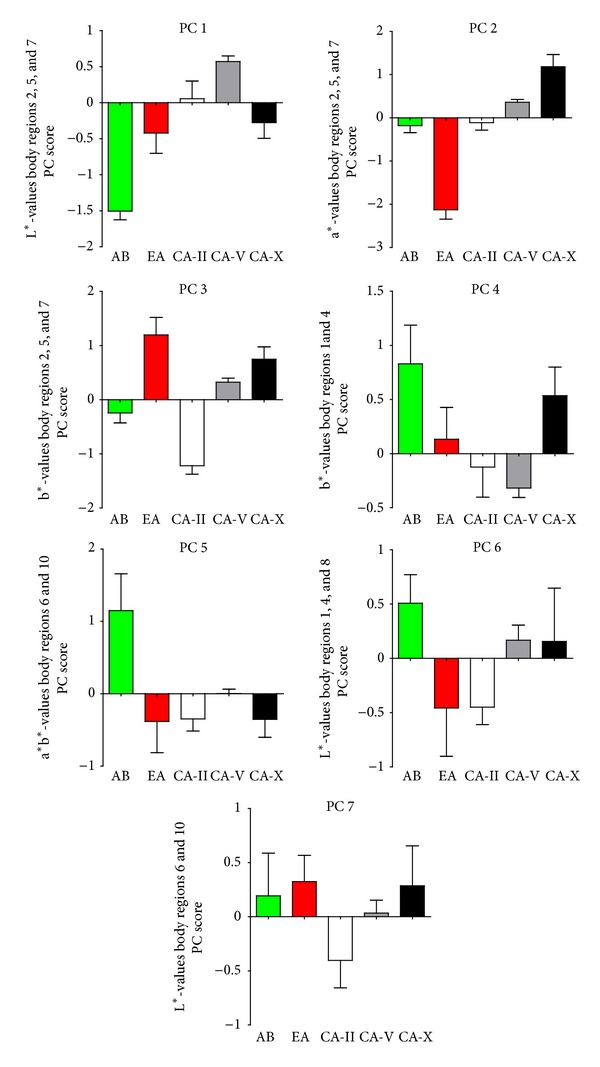
*Principal components analysis (PCA).* Shown are mean (±SE) principal component scores extracted from PCA on L*a*b* values of all 10 regions on the male body surface for each population. The strongest axis loadings (see main text) are further indicated. Arroyo Bonita (AB, green square), El Azufre (EA, red square), Cueva del Azufre chamber II (CA-II, white square), chamber V (CA-V, grey square), and chamber X (CA-X, black square).

As a qualitative overview, we present mean values (±SE) for L*, a*, and b* of the different spots for all populations examined (Figure 3). However, our central question was whether the examined populations differ overall in body colouration and which colour values of the particular spots contributed most to possible differences. We thus conducted PCA (principle component analysis) to condense our data. L*a*b*-values from all 10 spots were included, and PC axes with eigenvalues above 1 were extracted. Resulting axis loadings were Varimax-rotated for better interpretation. For presentation purpose, mean (±SE) PC scores with strongest axis loadings (values that were within a range of 0.10 starting at the highest value) are shown at the respective axes (Figure 4). PC scores were then used as dependent variables in MANCOVA with “population” as fixed factor and body length of the photographed fish (“SL”) as covariate, including the interaction term “population × SL”. *Post hoc* ANCOVAs for each extracted PC axis separately (but otherwise identical model structure) were applied to disentangle which PC axes contributed to a possible population effect in the MANCOVA. Interaction terms were removed if nonsignificant. Significant effects of “SL” were *post hoc* analysed using Pearson correlations while in case of significant interaction terms standardized residuals were analysed instead. All analyses were conducted using SPSS 13 and all data are presented as mean ± S.E. Prior to all analyses, data were checked to meet requirements of normal distribution and homoscedasticity.

We calculated the relative distension of the abdomen of each male (ratio between maximum body height and standard length). A previous study established this as an estimate of males' nutritional state [73]. Ratios were compared between populations using one-way ANOVA, and Fisher's LSD tests were applied for pairwise *post hoc* comparisons. 

### 2.2. Preferences of Surface Females for Resident over CA Males

In dichotomous association preference tests we presented animations of resident and cave males (CA-II) to lab-reared surface females from RO and EA.

#### 2.2.1. General Testing Procedure

To produce the video animations, we used male photographs from the analysis of body colouration (after calibration; AB: *N* = 12; EA: *N* = 9; CA-II: *N* = 22). From each picture, the image of the male was extracted from the background using the “magic extractor” function in Adobe Photoshop CS5. The resulting images were then animated and converted into an html file (resolution 800 × 600, 30 frames per second) using Adobe Flash Professional CS5. 

 A straight movement of the pictures from left to right and right to left was generated in front of a uniformly light grey background. The animations were 12 s long: twice 5 s for the distance of 30 cm on the screen forth and back, each followed by an invisible turn of 1 s (see also [64] for a detailed description of the method). Simultaneous playback was performed using two identical computer monitors (Samsung SyncMaster P2470LHD) with a dual-head graphic card. The monitor refresh rate was 85 Hz, and the html animations were run in infinite loops during the experiment using Mozilla Firefox 3.

For the preference tests, monitors were placed on either side of a test tank (60 × 30 × 30 cm) that was visually divided into three sections: two preference zones (10 cm length) adjacent to the monitors and a central neutral zone (40 cm length). Both long sides of the tank were covered by black plastic foil, so the focal fish did not see out of the test tank. The test tank was filled with aged tap water to a level of 25 cm, which was also the height of the monitors. Water temperature was maintained at 27-28°C, and illumination was provided through two 100 Watt neon tubes on the ceiling of the experimental room. The water of the test tank was aerated between trials, but the air pump was turned off during the experiment. We observed the focal female via a web cam (Microsoft LifeCam VX-2000) that was fixed in a central position approximately 70 cm above the test tank. 

To initiate a trial, we introduced a single focal female either from RO (*N* = 16; SL = 43.4 ± 1.6 mm) or from EA (*N* = 19; SL = 35.7 ± 2.1 mm) into the test tank and started the video playback. After a habituation period of 5 minutes, we started a 5-minute observation period during which we measured association times, that is, times spent in each preference zone. RO focal females were tested with animations of an AB male on one side and of a CA-II male on the other side, while EA females were presented with animations of EA and CA-II males. Male size on screen was set to 30 mm and all males were taken from the larger male size class (>40 cm SL), which is brightly coloured in surface populations (see Results). To account for potential side-biases, we interchanged the stimulus animations and repeated measurement of female preferences after another 5 minutes of habituation. We decided a priori to assume side bias if a female spent 80% of its association time during both test units in one of the two preference zones. Furthermore, we discarded trials in which females spent less than 50% of their time in the preference zones due to low response. Based on these criteria two trials with RO females and 6 trials with EA females had to be discarded. After a trial, we summed the times individual females spent near either male type during the two test units and measured female body size (SL).

#### 2.2.2. Statistical Analysis

Times focal females spent near the resident male or the CA-II male were compared using paired samples *t*-tests. As reinforcement theory predicts stronger conspecific mate preference in populations that inhabit adjacent habitats (if population hybrids have a reduced fitness [70]), we compared the strength of preference [SOP, (time spent with own male−time spent with CA-II male)/total choice time] between both focal female populations using ANCOVA. We included “focal female population” as a fixed factor and “focal female size (SL)” as a covariate as well as their interaction term in our initial model. However, the covariate and its interaction had no significant effects (*F* ≤ 0.30, *P* ≥ 0.65), and, thus, were removed from the final model.

### 2.3. Preferences of CA Females

In another experiment we asked whether CA females prefer their own male phenotype over EA males. We tested CA females from chamber V directly on site (in a perpetually dark area of chamber V) using a mobile test tank and an infrared nightshot goggle (Newton NV2x24 14061) emitting wavelengths above 850 nm, to which the visual pigments of *P. mexicana* are not sensitive [46]. 

#### 2.3.1. General Testing Procedure

Test females collected in cave chamber V were isolated from males for at least 24 h in a perforated Sterilite container (62-L), half submerged at a shallow bank in chamber V. Stimulus males were acclimated in another container. To simulate a migration event, EA males were caught in EA just outside the cave and brought directly into the cave for immediate use as stimuli. We used another Sterilite container as our mate choice arena, which we placed into the water current in a way that the water level inside the container was maintained at 15 cm. We divided the test arena visually into three equal-sized compartments by laying small stones at the bottom of the container; the two outer zones thus became the preference zones. We then placed a plastic wire-mesh cylinder (5 mm mesh-size, 12 cm diameter) into each of the outer compartments. These eventually held the stimulus males, allowing females to choose by non-visual cues. We measured the time females spent in each of the outer preference zones (i.e., in the proximity of a male). To accomplish this in total darkness, the experimenter was observing the focal female through the nightshot goggle and announced when the female would enter or leave a compartment (“left out,” “left in,” “right out,” and “right in”) to another person who was sitting about 5 m away and operated two stop watches (one for each compartment) that were both equipped with internal light diodes. This procedure ensured that choice tests were carried out under completely dark conditions. However, to prevent errors due to mishandling of stopwatches or the nightshot google, all experimenters were thoroughly trained prior to the tests. In our first experiment, we sought to validate our experimental approach by asking whether females exhibit a preference for large male body size as shown beforehand (e.g., [35]). As predicted, females preferred the larger (30.9 ± 0.4 mm; 302 ± 25 s) over the smaller stimulus male (24.7 ± 0.6 mm; 194 ± 21 s; paired samples *t*-test; *t*_14_ = 2.43, *P* = 0.029), indicating that our choice tests appropriately evaluated CA females' mating preferences.

In our main experiment, a CA-V male (27.8 ± 0.5 mm) and an equal-sized EA male (27.9 ± 0.7 mm; paired samples *t*-test; *t*_15_ = 0.31, *P* = 0.75) were gently transferred into the cylinders. Afterwards a focal female (36.4 ± 1.1 mm) was transferred into the central compartment. Measurement of association times commenced once the female started to swim freely. Association times were then recorded for 5 minutes, after which the two cylinders were carefully interchanged and measurement of association times was repeated. We checked for side-bias and low response (see above), but no trial had to be discarded based on those criteria.

#### 2.3.2. Statistical Analysis

Association times of the focal females with either male type were compared using a paired samples *t*-test. We tested for possible effects of female SL by calculating Spearman's rank order correlation between SOP values (see above) and focal female SL.

## 3. Results

### 3.1. Differences in Male Colouration along the Light/Dark Gradient

**Figure 5:**
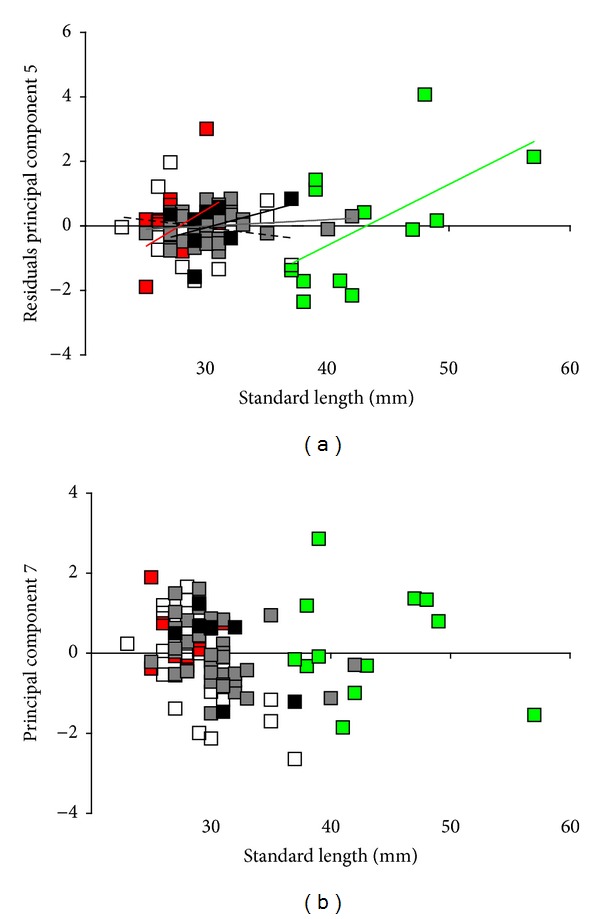
*Correlations of PC scores and male body size (SL)*. (a) Body size dependence of residuals obtained from ANCOVA on scores from PC5. A significant correlation was found only in surface AB males (results from Pearson's correlation: AB: *r*_*p*_ = 0.58, *P* = 0.05; EA: *r*_*p*_ = 0.34, *P* = 0.37; CA-II: *r*_*p*_ = −0.18, *P* = 0.42; CA-V: *r*_*p*_ = 0.15, *P* = 0.35; CA-X: *r*_*p*_ = 0.39, *P* = 0.34). (b) Body size dependence of PC 7. Arroyo Bonita (AB, green square), El Azufre (EA, red square), Cueva del Azufre chamber II (CA-II, white square), chamber V (CA-V, grey square), and chamber X (CA-X, black square).

PCA extracted seven components with eigenvalues >1 (Figure 4), accounting for 80.81% of the total variance explained. MANCOVA on those PC scores detected significant effects of the covariate “SL” and the interaction term “population × SL” (Table 2(a)). The latter suggests that a considerable portion of the total variance can be ascribed to population differences in the relationships between male body size and body colouration; below we discuss this effect for PC5 (see Figure 5(a)). 

*Post hoc* ANCOVAs on the seven PCs, however, found support for major population differences, as the main factor “population” was significant for PC 1, 2, 3, and 5, while “SL” had a significant effect in the analysis of PC 7, and the interaction term “population × SL” was significant for PC 5 (Table 2(b)). 

PC 1 received the strongest loadings from the L*-values of body regions 2, 5, and 7 and separates AB males from all other populations (Figure 4(a)), as AB males had lower L*-values, that is, darker dorsal regions (Figures 2(a) and 3). PC 2 received the strongest loadings from a*-values of regions 2, 5, and 7 separating EA males from all other populations (Figure 4(b)). EA males exhibited a somewhat green-shifted dorsal region (Figure 3). PC 3 received strongest loadings from b*-values of regions 2, 5, and 7 and separates CA-II and EA males from all other populations, indicating that CA-II males had more bluish and EA males more yellowish dorsal regions (Figure 3). 

PC 5 received the strongest loadings from a* and b*-values of regions 6 and 10, separating males of both surface populations from all CA males (Figure 4). Surface males had more yellowish dorsal and caudal fin margins, while cave molly males were nearly colourless at these regions (Figure 3). However, also a significant effect of the interaction term “population × SL” was uncovered, suggesting slope heterogeneity in the relationship with male SL, and population-wise *post hoc* Pearson's correlations found a significant positive correlation between SL and standardized residuals only in AB males (Figure 5(a)). Hence, larger males in this population had significantly more yellowish dorsal and caudal fin margins than smaller ones while no such effect was found in the other populations (see also Figure 3). 

A significant effect of the covariate “SL” was found in the ANCOVA on PC 7, and Pearson correlation uncovered a significant negative correlation between PC scores and male body length (*r*_*p*_ = −0.291, *P* = 0.005, *N* = 91; Figure 5(b)). As PC 7 received the strongest axis loadings from lightness (L*) of regions 6 and 10; the negative correlation suggests that larger males have darker dorsal and caudal fin margins than smaller ones. 

### 3.2. Male Nutritional State Estimated from Abdominal Distension

**Figure 6:**
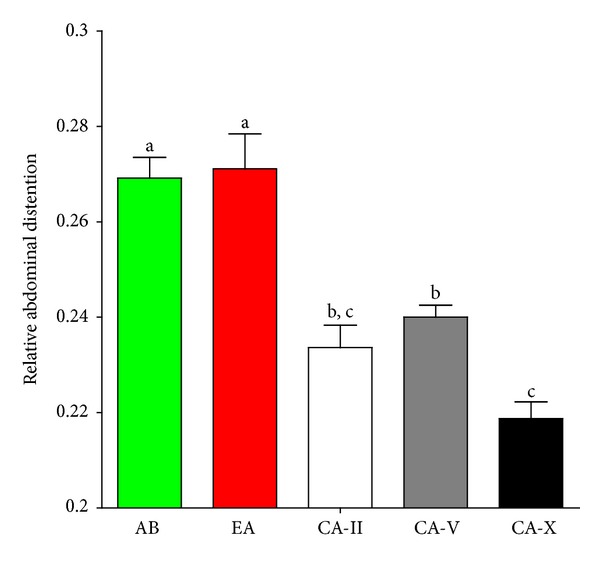
*Nutritional state estimated through male abdominal distension.* Abdominal distension was calculated as the body height/length ratio (see main text). Shown are means (±SE) for males from Arroyo Bonita (AB, green square), El Azufre (EA, red square), Cueva del Azufre chamber II (CA-II, white square), chamber V (CA-V, grey square), and chamber X (CA-X, black square). Significant differences (based on *post hoc* LSD tests) are indicated by different letters.

One-way ANOVA uncovered significant population differences in male abdominal distension (*mean square* = 6.45, *F*_4,90_ = 17.93, *P* < 0.001), and *post hoc* pair-wise LSD comparisons found both surface populations to have greater abdominal distensions than CA males (Figure 6). Within the cave, CA-X males differed significantly from all other populations (Figure 6).

### 3.3. Preferences of Surface Females for Resident and CA Males

**Figure 7:**
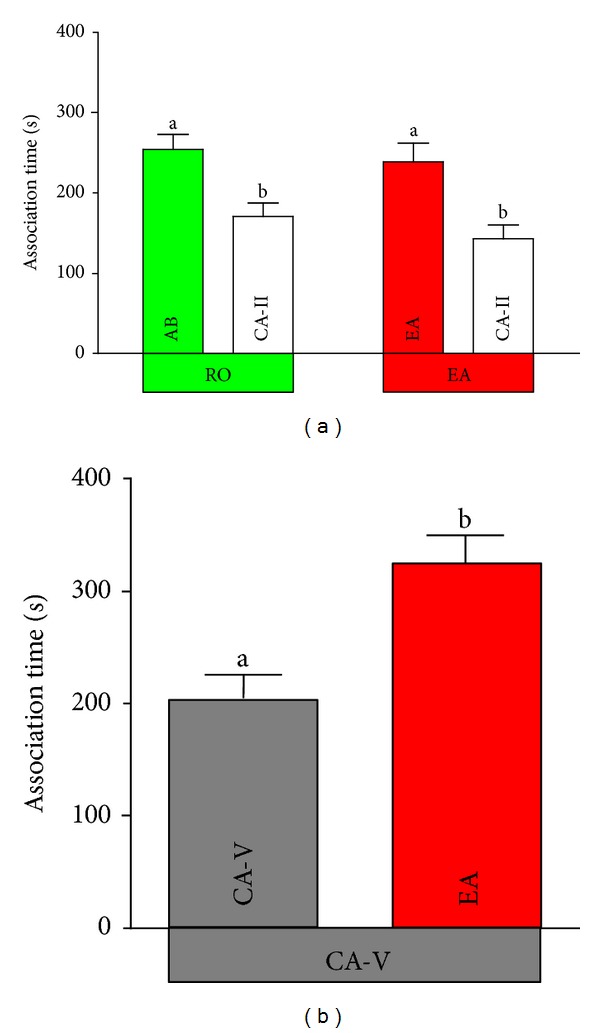
*Female association times near resident males and males from an alien population.* (a) Times females from the Río Oxolotán population (RO, green square) and the El Azufre (EA, red square) spent near animated resident males or a cave-adapted male (CA-II, white square). (b) Time CA-V (grey square) females spent near resident males or males from the El Azufre (EA) when tested in the cave using infrared techniques. Different letters indicate significant differences in paired samples *t*-tests.

Females from both populations examined spent significantly more time near animations showing the resident male phenotype (RO: *t*_13_ = 2.60, *P* = 0.022; EA: *t*_12_ = 2.38, *P* = 0.035; Figure 7(a)). When comparing the SOP between both populations no significant difference was detected (ANCOVA: *mean square *= 0.01, *F*_1,25_ = 0.10, *P* = 0.75).

### 3.4. Preferences of CA Females for Resident and EA Males

Contrary to prediction, CA-V females spent significantly more time with EA males than with males from their own population (*t*_15_ = 2.41, *P* = 0.029; Figure 7(b)). We found no significant correlation between focal female body size (SL) and SOP-values (Spearman's rank order correlation: *r*_*s*_ = 0.27, *P* = 0.31, *N* = 16). 

## 4. Discussion 

Our study reveals distinct differences in male body and fin colouration between *P. mexicana* males from ecologically divergent habitats. Surface males (AB)—living in an environment with numerous visual predators of mollies [17, 36, 75]—show pronounced countershading (a dark back and a silvery whitish ventral side) as a means to camouflage themselves from predators [76–79]. Piscine predators are largely absent from sulphidic waters [17, 36], but the hypoxia in sulphidic waters forces *P. mexicana* to the surface where they engage in aquatic surface respiration [33, 34]. Hence, *P. mexicana* in sulphidic surface habitats experience an up to twentyfold increased bird predation [69]. Still, countershading does not provide camouflage in this habitat type, as fish will be perceived by avian predators against the whitish background of the sulphide-and sulphate-rich water, and accordingly, countershading was reduced in EA males. A brighter dorsal side in EA fish may still be interpreted as a form of crypsis, enabling the prey species to resemble the typical visual background in their habitat [80–82].

Males from the Cueva del Azufre were generally far less pigmented and—with the exception of males from cave chamber II—had a uniform, pale appearance. Several body regions showed a slight shift toward a more reddish colouration, which can be readily explained by the lack of body pigmentation making capillary blood visible through the skin. Loss of body pigmentation is a typical feature of cave animals [83]. For example, pale and eyeless cave populations of the Mexican tetra *Astyanax mexicanus* (Characidae) became a model organism for EvoDevo studies on the evolution of troglomorphic characters (for a review see [26]). In *A. mexicanus*, eye loss appears to be mainly driven by pleiotropic antagonistic selection, as eye reduction is coupled with an improvement of the gustatory sense [28, 84, 85], whereas the loss of pigmentation is likely caused by the accumulation of selectively neutral mutations in the absence of stabilizing selection [29, 86]. Colour genes are rather well understood in poeciliids (see, e.g., [87]), and attempts to identify the molecular basis of trait evolution in *P. mexicana* ecotypes—including genes responsible for colour ornamentation—are currently underway [88].

We detected a cline-like change in body colouration in the Cueva del Azufre, as males from cave chamber II, which receives some dim light from skylights, had slightly darker dorsal and caudal fin margins, and a slightly darker general body colouration than males from chambers V and X. Also, dorsal colouration in CA-II males was shifted towards bluer colouration. Three different hypotheses seek to explain gradual patterns of morphological differentiation among different cave chambers in the Cueva del Azufre. (1) For many decades it was believed that the morphocline in eye size is generated and maintained through intensive gene flow promoted by migration from the adjacent surface waters into the cave and migration or passive drift *vice versa *[31, 47, 57]. (2) More recent population genetic analyses [48, 65], however, found reduced gene flow among cave chambers and a weak, unidirectional gene flow out of the cave. Thus, continuous population hybridization can be ruled out as an explanation. Fontanier and Tobler [43], therefore, formulated two alternative, not mutually exclusive hypotheses. (2a) First, fish may be locally adapted to the particular environmental conditions of a given cave chamber (e.g., different levels of light and/or H_2_S concentrations) even though the spatial scale at which adaptation occurs is extremely small (i.e., over few dozen to hundred meters) and no physical barriers to migration exist, the sole exception being a small water fall separating the hindmost chamber XIII from adjacent chambers. (2b) Differences among cave chambers could also be explained by phenotypic plasticity [89], including potential epigenetic inheritance of different light-induced trait expression [90]. 

Larger surface-(AB and EA), but not cave-adapted males (CA) exhibited colour ornaments that likely function in intra- and intersexual selection, namely, orange/yellow dorsal and caudal fin margins. Parzefall [55] proposed that these colourful fin margins represent a feature that typically characterizes dominant males. As dominance in *P. mexicana* is related to body size [13], our results, therefore, largely support this earlier notion. In combination with other morphological features separating surface and cave fish [2], differences in colouration seem to play a crucial role in promoting mating preferences of surface females in favour of males from their own ecotype. Indeed, a recent study found RO females to prefer animations showing males with artificially increased colouration over animations with artificially reduced colouration [64]. Behavioural differences between surface and cave-adapted *P. mexicana* males—like reduced sexual activity [37, 91], reduced shoaling [47, 92], and reduced aggression [13, 55, 56]—were ruled out as a source of information for focal females by our experimental approach as we used video animated images as stimuli; still, divergent behaviour of CA males may play an additional role in female mate choice in nature, which ought to lead to an even stronger rejection by surface females. 

One of our initial predictions was that reinforcement could play a role if population hybrids have a reduced fitness [70], in which case contypic mate preferences should be stronger in populations from adjacent habitats (i.e., EA females should have stronger preferences than AB females). Our data do not support this idea, suggesting that the visual female preference of surface females for more colourful (and better nourished) males did not evolve under specific selection to avoid unfit hybrid offspring. Rather our results suggest that the widespread female preference for male sexual ornaments of *P. mexicana* females [64] explains this pattern, which is a result of cave adapted males having reduced such ornaments.

We argue that phenotype-assortative mate choice of EA (and AB) females further promotes genetic differentiation between surface and cave ecotypes in the Cueva del Azufre system by selecting against potential migrant males that leave the cave habitat, thus fostering ecological speciation along the light-dark interface [16]. Colour differences not only put migrant CA males at a disadvantage in sexual selection, but—along with reduced predator evasion abilities due to maladapted sensory systems [68]—ought to translate into an increased bird predation rate. In a study by Tobler et al. [8], CA females from chamber II were tested for their visual preferences for resident over EA males and preferred resident males. CA females preferred resident male phenotypes, suggesting that some level of phenotype-assortative mate choice occurs where light penetrates the cave. However, our present study demonstrates that CA females do not exhibit such a preference in the deeper, perpetually dark areas, as CA-V females did not prefer the resident male phenotype in our infrared-based experiment. Female mate choice, therefore, probably plays a subordinate role in maintaining genetic differentiation when considering the potential migration route from the outside into the cave, and other selection factors likely play a more important role. For instance, surface females (AB) are not able to complete their reproductive life cycle in darkness [10], probably because melatonin production by the epiphysis is not suppressed by light, thus suppressing the release of sexual hormones (see [93] for a review).

Cave molly females in our infrared-based mate choice experiment under field conditions discriminated in favour of alien (EA) males. We argue that this reflects the previously reported preference of CA females for well-nourished males, given that body condition reliably indicates male fitness in extreme habitats [73]. This alludes to a conflict between a sexually selected trait and the recognition of locally adapted males (in other studies on more distantly related taxa, this would be termed the species recognition component of mate choice, see [94, 95]). Our results support this view as EA males were better nourished (based on abdominal distension) than CA males from all three chambers investigated. The Cueva del Azufre has high primary production by sulphide-oxidizing bacteria, which constitute the magnitude of the diet of the inhabiting fish [39], and fish densities locally exceed 100–200 individuals per m^2^ compared to 2–50 individuals per m^2^ in El Azufre [47]. Strong resource competition, along with the relatively low nutritional value of the sulphur bacteria [39], may explain the tremendously low body condition of CA males [41, 42]. Another point that should be addressed in future studies is the role of chemical cues in mate choice in this system as chemical cues have been shown to play an important role for mate choice in poeciliid fishes (see [96, 97] for review). For example, *Xiphophorus birchmanni* females are able to select well-nourished males based upon chemical cues [98]. However, it remains unclear what effect the presence of H_2_S would have on such chemically mediated cues. For example, studies in the poeciliid genus *Xiphophorus* suggest that disruption of pheromone-based female choice may be caused by (anthropogenic) water pollution [99]. 

Even though we are currently lacking the data to prove this, we suggest that natural selection by the cave environment—including the necessity for food acquisition in darkness despite increased competition—imposes a so strong disadvantage on immigrating fish that only very few mature surface males will ever be present in the cave environment, precluding the evolution of a stronger ecotype-assortative mating preference in CA females [100, 101].

## Figures and Tables

**Table 1:** Body length (SL) and sample sizes (*N*) of males used for the analyses of body colouration and general habitat properties in terms of light availability and turbidity.

Population origin	*N *	Standard length ± SE	Light availability	Turbidity [NTU]
Arroyo Bonita (AB)	12	42.8 ± 1.8	++	0.0
El Azufre (EA)	9	27.8 ± 0.73	++	67.0–104.3
Cueva del Azufre (CA-II, chamber II)	22	28.9 ± 0.7	o	81.4
Cueva del Azufre (CA-V, chamber V)	40	30.0 ± 0.5	−	45.3–85.0
Cueva del Azufre (CA-X, chamber X)	8	30.1 ± 1.0	−−	39.0–74.6

Light availability is defined as ++: regular surface waters; +: surface water with reduced light availability due to high turbidity; o: cave habitat with dim light available through skylights; −: cave habitat with dim light available only in some areas; −−: perpetually dark cave habitat. Turbidity was measured using a shuttered turbidity probe in nephelometric turbidity units (NTU); data were taken from Tobler et al. (2006) [17].

**Table:** (a)

	Wilk's *λ*	*F*	df (error df)	*P*	Partial eta²
Population	0.70	1.01	28 (271.83)	0.45	0.086
SL	**0.72**	**4.14**	**7 (75)**	**0.001**	**0.28**
Population × SL	**0.56**	**1.70**	**28 (271.83)**	**0.018**	**0.14**

**Table:** (b)

	Mean square	*F*	df	*P*	Partial eta²
Principal component 1					
Population	**6.312**	**11.320**	**4**	**0.0001**	**0.348**
SL	0.030	0.054	1	0.816	0.001
Error	0.56		85		

Principal component 2					
Population	**14.743**	**40.465**	**4**	**0.0001**	**0.656**
SL	1.237	3.396	1	0.069	0.038
Error	0.36		85		

Principal component 3					
Population	**13.714**	**33.363**	**4**	**0.0001**	**0.611**
SL	0.013	0.032	1	0.858	0.000
Error	0.41		85		

Principal component 4					
Population	2.151	2.444	4	0.053	0.103
SL	0.142	0.162	1	0.688	0.002
Error	0.88		85		

Principal component 5					
Population	**1.893**	**2.788**	**4**	**0.032**	**0.121**
SL	**3.802**	**5.600**	**1**	**0.020**	**0.065**
Population × SL	**2.142**	**3.155**	**4**	**0.018**	**0.135**
Error	0.70		81		

Principal component 6					
Population	1.510	1.635	4	0.173	0.071
SL	0.725	0.785	1	0.378	0.009
Error	0.92		85		

Principal component 7					
Population	**3.869**	**4.551**	**4**	**0.002**	**0.176**
SL	**12.051**	**14.175**	**1**	**<0.001**	**0.143**
Error	0.85		85		

## References

[1] Doebeli M, Dieckmann U (2003). Speciation along environmental gradients. *Nature*.

[2] Tobler M, DeWitt TJ, Schlupp I (2008). Toxic hydrogen sulfide and dark caves: phenotypic and genetic divergence across two abiotic environmental gradients in *Poecilia mexicana*. *Evolution*.

[3] Endler JA (1986). *Natural Selection in the Wild*.

[4] Hendry AP (2004). Selection against migrants contributes to the rapid evolution of ecologically dependent reproductive isolation. *Evolutionary Ecology Research*.

[5] Nosil P, Vines TH, Funk DJ (2005). Perspective: reproductive isolation caused by natural selection against immigrants from divergent habitats. *Evolution*.

[6] Grahame JW, Wilding CS, Butlin RK (2006). Adaptation to a steep environmental gradient and an associated barrier to gene exchange in *Littorina saxatilis*. *Evolution*.

[7] Nosil P (2004). Reproductive isolation caused by visual predation on migrants between divergent environments. *Proceedings of the Royal Society B*.

[8] Tobler M, Riesch R, Tobler CM, Schulz-Mirbach T, Plath M (2009). Natural and sexual selection against immigrants maintains differentiation among micro-allopatric populations. *Journal of Evolutionary Biology*.

[9] Tobler M, Palacios M, Chapman LJ (2011). Evolution in extreme environments: replicated phenotypic differentiation in livebearing fish inhabiting sulfidic springs. *Evolution*.

[10] Riesch R, Plath M, Schlupp I (2011). Speciation in caves: experimental evidence that permanent darkness promotes reproductive isolation. *Biology Letters*.

[11] Via S, Bouck AC, Skillman S (2000). Reproductive isolation between divergent races of pea aphids on two hosts. II. Selection against migrants and hybrids in the parental environments. *Evolution*.

[12] van Doorn GS, Edelaar P, Weissing FJ (2009). On the origin of species by natural and sexual selection. *Science*.

[13] Bierbach D, Klein M, Sassmannshausen V (2012). Divergent evolution of male aggressive behaviour: another reproductive isolation mechanism in extremophile poeciliid fishes. *International Journal of Evolutionary Biology*.

[14] Plath M, Riesch R, Oranth A (2010). Complementary effect of natural and sexual selection against immigrants maintains differentiation between locally adapted fish. *Naturwissenschaften*.

[15] Snowberg LK, Benkman CW (2009). Mate choice based on a key ecological performance trait. *Journal of Evolutionary Biology*.

[16] Plath M, Pfenninger M, Lerp H (2013). Genetic differentiation and selection against migrants in evolutionarily replicated extreme environments. *Evolution*.

[17] Tobler M, Schlupp I, Heubel KU (2006). Life on the edge: hydrogen sulfide and the fish communities of a Mexican cave and surrounding waters. *Extremophiles*.

[18] Calow P (1989). Proximate and ultimate responses to stress in biological systems. *Biological Journal of the Linnean Society*.

[19] Rothschild LJ, Mancinelli RL (2001). Life in extreme environments. *Nature*.

[20] Bagarinao T (1992). Sulfide as an environmental factor and toxicant: tolerance and adaptations in aquatic organisms. *Aquatic Toxicology*.

[21] Grieshaber MK, Völke S (1998). Animal adaptations for tolerance and exploitation of poisonous sulfide. *Annual Review of Physiology*.

[22] Yoshizawa M, Gorički Š, Soares D, Jeffery WR (2010). Evolution of a behavioral shift mediated by superficial neuromasts helps cavefish find food in darkness. *Current Biology*.

[23] Jeffery WR, Strickler AG, Yamamoto Y (2003). To see or not to see: evolution of eye degeneration in Mexican blind cavefish. *Integrative and Comparative Biology*.

[24] Espinasa L, Yamamoto Y, Jeffery WR (2005). Non-optical releasers for aggressive behavior in blind and blinded *Astyanax* (Teleostei, Characidae). *Behavioural Processes*.

[25] Tobler M, Schlupp I, Plath M (2008). Does divergence in female mate choice affect male size distributions in two cave fish populations?. *Biology Letters*.

[26] Jeffery WR (2001). Cavefish as a model system in evolutionary developmental biology. *Developmental Biology*.

[27] Klaus S, Mendoza JCE, Liew JH, Plath M, Meier R, Yeo DCJ (2013). Rapid evolution of troglomorphic characters suggests selection rather than neutral mutation as a driver of eye reduction in cave crabs. *Biology Letters*.

[28] Jeffery WR (2005). Adaptive evolution of eye degeneration in the Mexican blind cavefish. *Journal of Heredity*.

[29] Protas M, Conrad M, Gross JB, Tabin C, Borowsky R (2007). Regressive evolution in the Mexican cave tetra, *Astyanax mexicanus*. *Current Biology*.

[30] Aden E, Culver D, White W (2005). Adaptation to darkness. *Encyclopedia of Caves*.

[31] Gordon MS, Rosen DE (1962). A cavernicolous form of the poeciliid dish *Poecilia sphenops* from Tabasco, México. *Copeia*.

[32] Tobler M, Riesch R, de León FJG, Schlupp I, Plath M (2008). A new and morphologically distinct population of cavernicolous *Poecilia mexicana* (Poeciliidae: Teleostei). *Environmental Biology of Fishes*.

[33] Plath M, Tobler M, Riesch R, de León FJG, Giere O, Schlupp I (2007). Survival in an extreme habitat: the roles of behaviour and energy limitation. *Naturwissenschaften*.

[34] Tobler M, Riesch RW, Tobler CM, Plath M (2009). Compensatory behaviour in response to sulphide-induced hypoxia affects time budgets, feeding efficiency, and predation risk. *Evolutionary Ecology Research*.

[35] Plath M, Parzefall J, Körner KE, Schlupp I (2004). Sexual selection in darkness? Female mating preferences in surface- and cave-dwelling Atlantic mollies, *Poecilia mexicana* (Poeciliidae, Teleostei). *Behavioral Ecology and Sociobiology*.

[36] Riesch R, Duwe V, Herrmann N (2009). Variation along the shy-bold continuum in extremophile fishes (*Poecilia mexicana*, *Poecilia sulphuraria*). *Behavioral Ecology and Sociobiology*.

[37] Plath M (2008). Male mating behavior and costs of sexual harassment for females in cavernicolous and extremophile populations of Atlantic mollies (*Poecilia mexicana*). *Behaviour*.

[38] Tobler M (2008). Divergence in trophic ecology characterizes colonization of extreme habitats. *Biological Journal of the Linnean Society*.

[39] Roach KA, Tobler M, Winemiller KO (2011). Hydrogen sulfide, bacteria, and fish: a unique, subterranean food chain. *Ecology*.

[40] Riesch R, Plath M, de León FJG, Schlupp I (2010). Convergent life-history shifts: toxic environments result in big babies in two clades of poeciliids. *Naturwissenschaften*.

[41] Riesch R, Plath M, Schlupp I (2010). Toxic hydrogen sulfide and dark caves: life-history adaptations in a livebearing fish (*Poecilia mexicana*, Poeciliidae). *Ecology*.

[42] Riesch R, Plath M, Schlupp I (2011). Toxic hydrogen sulphide and dark caves: pronounced male life-history divergence among locally adapted *Poecilia mexicana* (Poeciliidae). *Journal of Evolutionary Biology*.

[43] Fontanier ME, Tobler M (2009). A morphological gradient revisited: cave mollies vary not only in eye size. *Environmental Biology of Fishes*.

[44] Tobler M, Culumber ZW, Plath M, Winemiller KO, Rosenthal GG (2011). An indigenous religious ritual selects for resistance to a toxicant in a livebearing fish. *Biology Letters*.

[45] Bierbach D, Schleucher E, Hildenbrand P (2010). Thermal tolerances in mollies (*Poecilia* spp.): reduced physiological fl exibility in stable environments?. *Bulletin of Fish Biology*.

[46] Körner KE, Schlupp I, Plath M, Loew ER (2006). Spectral sensitivity of mollies: comparing surface- and cave-dwelling Atlantic mollies, *Poecilia mexicana*. *Journal of Fish Biology*.

[47] Parzefall J (2001). A review of morphological and behavioural changes in the cave molly, *Poecilia mexicana*, from Tabasco, Mexico. *Environmental Biology of Fishes*.

[48] Plath M, Hauswaldt JS, Moll K (2007). Local adaptation and pronounced genetic differentiation in an extremophile fish, *Poecilia mexicana*, inhabiting a Mexican cave with toxic hydrogen sulphide. *Molecular Ecology*.

[49] Donohue I, Molinos JG (2009). Impacts of increased sediment loads on the ecology of lakes. *Biological Reviews*.

[50] Engström-Öst J, Candolin U (2007). Human-induced water turbidity alters selection on sexual displays in sticklebacks. *Behavioral Ecology*.

[51] Sundin J, Berglund A, Rosenqvist G (2010). Turbidity hampers mate choice in a pipefish. *Ethology*.

[52] Heubel KU, Schlupp I (2006). Turbidity affects association behaviour in male *Poecilia latipinna*. *Journal of Fish Biology*.

[53] Tobler M, Coleman SW, Perkins BD, Rosenthal GG (2010). Reduced opsin gene expression in a cave-dwelling fish. *Biology Letters*.

[54] Farnworth M

[55] Parzefall J (1969). Zur vergleichenden Ethologie verschiedener *Mollienesia*-Arten einschließlich einer Höhlenform von *Mollienesia sphenops*. *Behaviour*.

[56] Parzefall J (1974). Rückbildung aggressiver Verhaltensweisen bei einer Höhlenform von *Poecilia sphenops* (Pisces, Poeciliidae). *Zeitschrift für Tierpsychologie*.

[57] Peters N, Peters G, Parzefall J, Wilkens H (1973). Über degenerative und konstruktive Merkmale bei einer phylogenetisch jungen Höhlenform von *Poecilia sphenops* (Pisces, Poeciliidae). *Internationale Revue der Gesamten Hydrobiologie und Hydrographie*.

[58] Riesch R, Schlupp I, Langerhans RB, Plath M (2011). Shared and unique patterns of embryo development in extremophile poeciliids. *PLoS ONE*.

[59] Houde AE (1997). *Sex, Color, and Mate Choice in Guppies*.

[60] Grether GF, Kolluru GR, Nersissian K (2004). Individual colour patches as multicomponent signals. *Biological Reviews of the Cambridge Philosophical Society*.

[61] Price AC, Weadick CJ, Shim J, Rodd FH (2008). Pigments, patterns, and fish behavior. *Zebrafish*.

[62] Morris MR, Elias JA, Moretz JA (2001). Defining vertical bars in relation to female preference in the swordtail fish *Xiphophorus cortezi* (Cyprinodontiformes, Poeciliidae). *Ethology*.

[63] Morris MR, Mussel M, Ryan MJ (1995). Vertical bars on male *Xiphophorus multilineatus*: a signal that deters rival males and attracts females. *Behavioral Ecology*.

[64] Bierbach D, Jung CT, Hornung S, Streit B, Plath M (2013). Homosexual behaviour increases male attractiveness to females. *Biology Letters*.

[65] Plath M, Hermann B, Schröder C (2010). Locally adapted fish populations maintain small-scale genetic differentiation despite perturbation by a catastrophic flood event. *BMC Evolutionary Biology*.

[66] Hermes-Lima M, Zenteno-Savín T (2002). Animal response to drastic changes in oxygen availability and physiological oxidative stress. *Comparative Biochemistry and Physiology C*.

[67] Schlupp I, Colston TJ, Joachim BL, Riesch R (2013). Translocation of cave fish (*Poecilia mexicana*) within and between natural habitats along a toxicity gradient. *Ecology of Freshwater Fish*.

[68] Tobler M (2009). Does a predatory insect contribute to the divergence between cave- and surface-adapted fish populations?. *Biology Letters*.

[69] Riesch R, Oranth A, Dzienko J (2010). Extreme habitats are not refuges: poeciliids suffer from increased aerial predation risk in sulphidic southern Mexican habitats. *Biological Journal of the Linnean Society*.

[70] Sætre GP, Moum T, Bureš S, Král M, Adamjan M, Moreno J (1997). A sexually selected character displacement in flycatchers reinforces premating isolation. *Nature*.

[71] Heubel KU, Schlupp I (2008). Seasonal plasticity in male mating preferences in sailfin mollies. *Behavioral Ecology*.

[72] Plath M, Tobler M (2007). Sex recognition in surface- and cave-dwelling Atlantic molly females (*Poecilia mexicana*, Poeciliidae, Teleostei): influence of visual and non-visual cues. *Acta Ethologica*.

[73] Plath M, Heubel KU, de León FJG, Schlupp I (2005). Cave molly females (*Poecilia mexicana*, Poeciliidae, Teleostei) like well-fed males. *Behavioral Ecology and Sociobiology*.

[74] Parzefall J (1970). Morphologische Untersuchungen an einer Hohlenform von *Mollienesia sphenops* (Pisces, Poeciliidae). *Zeitschrift für Morphologie der Tiere*.

[75] Bierbach D, Schulte M, Herrmann N (2011). Predator-induced changes of female mating preferences: innate and experiential effects. *BMC Evolutionary Biology*.

[76] Rowland HM (2009). From Abbott Thayer to the present day: what have we learned about the function of countershading?. *Philosophical Transactions of the Royal Society B*.

[77] Thayer AH (1896). The law which underlies protective coloration. *The Auk*.

[78] Stevens M, Merilaita S (2009). Animal camouflage: current issues and new perspectives. *Philosophical Transactions of the Royal Society B*.

[79] Kiltie RA (1988). Countershading: universally deceptive or deceptively universal?. *Trends in Ecology and Evolution*.

[80] Endler JA (1983). Natural and sexual selection on color patterns in poeciliid fishes. *Environmental Biology of Fishes*.

[81] Endler J (1978). A predator's view of animal color patterns. *Evolutionary Biology*.

[82] Endler JA (1980). Natural selection on color patterns in *Poecilia reticulata*. *Evolution*.

[83] Culver D (1982). *Cave Life—Evolution and Ecology*.

[84] Yamamoto Y, Byerly MS, Jackman WR, Jeffery WR (2009). Pleiotropic functions of embryonic sonic hedgehog expression link jaw and taste bud amplification with eye loss during cavefish evolution. *Developmental Biology*.

[85] Yamamoto Y, Stock DW, Jeffery WR (2004). Hedgehog signalling controls eye degeneration in blind cavefish. *Nature*.

[86] Jeffery WR (2009). Regressive evolution in astyanax cavefish. *Annual Review of Genetics*.

[87] Schartl M, Walter RB, Shen Y (2013). The genome of the platyfish, *Xiphophorus maculatus*, provides insights into evolutionary adaptation and several complex traits. *Nature Genetics*.

[88] Kelley J, Passow C, Plath M, Rodriguez LA, Yee MC, Tobler M (2012). Genomic resources for a model in adaptation and speciation research: characterization of the *Poecilia mexicana* transcriptome. *BMC Genomics*.

[89] Pigliucci M (2001). *Phenotypic Plasticity: Beyond Nature and Nurture*.

[90] Goldberg AD, Allis CD, Bernstein E (2007). Epigenetics: a landscape takes shape. *Cell*.

[91] Plath M, Parzefall J, Schlupp I (2003). The role of sexual harassment in cave and surface dwelling populations of the Atlantic molly, *Poecilia mexicana* (Poeciliidae, Teleostei). *Behavioral Ecology and Sociobiology*.

[92] Plath M, Schlupp I (2008). Parallel evolution leads to reduced shoaling behavior in two cave dwelling populations of Atlantic mollies (*Poecilia mexicana*, Poeciliidae, Teleostei). *Environmental Biology of Fishes*.

[93] Vanecek J (1998). Cellular mechanisms of melatonin action. *Physiological Reviews*.

[94] Hankison SJ, Morris MR (2002). Sexual selection and species recognition in the pygmy swordtail, *Xiphophorus pygmaeus*: conflicting preferences. *Behavioral Ecology and Sociobiology*.

[95] Hankison SJ, Morris MR (2003). Avoiding a compromise between sexual selection and species recognition: female swordtail fish assess multiple species-specific cues. *Behavioral Ecology*.

[96] Sargent RC, Rush VN, Wisenden BD, Yan HY (1998). Courtship and mate choice in fishes: integrating behavioral and sensory ecology. *The American Zoologist*.

[97] Magurran AE (2005). *Evolutionary Ecology: The Trinidadian Guppy*.

[98] Fisher HS, Rosenthal GG (2006). Female swordtail fish use chemical cues to select well-fed mates. *Animal Behaviour*.

[99] Fisher HS, Wong BBM, Rosenthal GG (2006). Alteration of the chemical environment disrupts communication in a freshwater fish. *Proceedings of the Royal Society B*.

[100] Lenormand T (2012). From local adaptation to speciation: specialization and reinforcement. *International Journal of Ecology*.

[101] Servedio MR, Noor MAF (2003). The role of reinforcement in speciation: theory and data. *Annual Review of Ecology, Evolution, and Systematics*.

